# Experimental Design-Based Dispersive Liquid–Liquid Microextraction with GC-FID for Determination of Polycyclic Aromatic Hydrocarbons in Surface Water

**DOI:** 10.1021/acsomega.6c01489

**Published:** 2026-05-19

**Authors:** Chen Minghong, Song Peiyu, Tien Ping Lee, Garret Chan Zhe Ming, Philip J. Marriott, Yong Foo Wong

**Affiliations:** † Center for Research on Multidimensional Separation Science, School of Chemical Sciences, 377278 Universiti Sains Malaysia, Gelugor, 11800 USM Penang, Malaysia; ‡ 26696 The Royal College of Surgeons in Ireland and University College Dublin Malaysia Campus, 4 Jalan Sepoy Lines, George Town, 10450 Penang, Malaysia; § Australian Centre for Research on Separation Science, School of Chemistry, 2541 Monash University, Wellington Road, Clayton, Victoria 3800, Australia

## Abstract

Polycyclic aromatic hydrocarbons (PAHs), designated as persistent organic pollutants, are ubiquitous environmental contaminants extensively distributed across surface water ecosystems. This study applies multivariate optimization strategies to a dispersive liquid–liquid microextraction (DLLME) approach for the extraction of acenaphthene, fluorene, anthracene, phenanthrene, fluoranthene, pyrene, benzo­(a)­anthracene, and benzo­(a)­pyrene, followed by gas chromatography-flame ionization detection (GC-FID). Extraction selectivity and efficiency were systematically evaluated using extraction and dispersive solvents spanning a range of dielectric constants. A fractional factorial design (FrFD) was used to screen key DLLME variables, followed by Box-Behnken design (BBD) optimization. Separation of all target PAHs was achieved on a DB-5HT column with an analysis time of <19 min. The optimized method was linear over 5–200 μg/L, with limits of detection of 0.11–0.33 μg/L. Good precision was obtained with relative standard deviations of <10% for peak areas and <0.2% for retention times. The method achieved an AGREE greenness score of 0.57, enrichment factors of 19.6–48.1, and recoveries of 65–111%. The validated DLLME-GC-FID method was successfully applied to quantify PAHs in terrestrial waters (wastewater, lake, river, and drain water).

## Graphical abstract

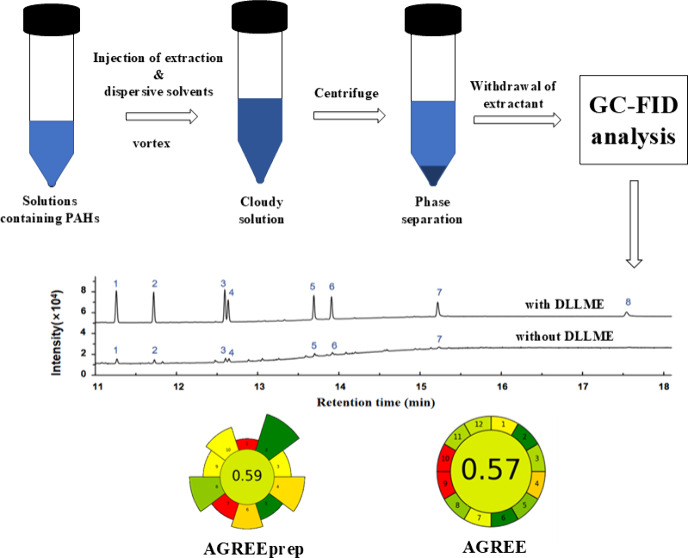

## 1 Introduction

Polycyclic aromatic hydrocarbons (PAHs) are organic pollutants containing two or more fused benzene rings and are widely associated with carcinogenic, teratogenic, and mutagenic properties.^1^ These hydrophobic substances can bioaccumulate with long environmental half-lives and are classified as high priority ecotoxicants of the first danger class.^1,2^ Anthropogenic activities (e.g., fossil fuel combustion, industrial processes, biomass burning, and others) are predominantly recognized as the primary sources of PAHs in most environmental compartments.^3–6^ In the present work, the hydrophobic nature of PAHs facilitates adsorption onto suspended particulate matter and organic material in aquatic systems, followed by deposition into underlying sediments.^7^ These persistent organic pollutants have been reported in surface waters as well as estuarine and coastal environments and can potentially disrupt ecosystem functioning.^7,8^

From a public health perspective, PAHs exposure via upon ingestion, has been linked to a spectrum of adverse health effects such as cancer and reduced fertility.^2,9^ Sixteen unsubstituted PAHs have been designated as priority pollutants by the United States Environmental Protection Agency (USEPA).^10^ The International Agency for Research on Cancer (IARC) has classified benzo­(a)­pyrene (BaP) in Group 1 (carcinogen to humans) and other PAHs in Group 2A (possible human carcinogens).^10,11^ The maximum permissible concentrations of benzo­(a)­pyrene (0.00001 mg/L) in water intended for drinking water is regulated by the European Union (EU). The EU limits the total content of polyaromatic compounds in drinking water to <0.1 μg/L.^12^ In heavily polluted waterways, PAH concentrations have been reported to reach 10 μg/mL.^13^ Epidemiological evidence also suggests a positive relationship between increased cancer incidence in human populations that are having high exposure to organic ecotoxins.^14^ For these reasons, quantitative assessments of PAH occurrence and distribution in surface waters remain important and fertile research topics.^15,16^

PAHs in water and environmental matrices are routinely analyzed using either gas chromatography (GC) and/or liquid chromatography (LC) with various detection modalities.^17,18^ The coupling of chromatography to mass spectrometry has been the preferred technique, owing to the distinct selectivity and sensitivity, particularly when operated in selected ion monitoring (SIM) or multiple reaction monitoring (SRM) modes.^19^ Additionally, LC coupled with fluorescence detection is also extensively utilized for PAH analysis.^20,21^ As PAHs typically occur at trace to ultratrace levels in complex real-world matrices, sample pretreatment and/or preconcentration is generally required prior to instrumental analysis.^22,23^ Conventional approaches such as liquid–liquid extraction and solid-phase extraction are widely used but are often solvent-intensive and laborious and generate substantial hazardous waste. More recently, microextraction techniques (e.g., dispersive microsolid phase extraction, dispersive liquid–liquid microextraction (DLLME), solid phase microextraction, etc.) have emerged as attractive alternatives, providing advantages such as speed and simplicity and requiring substantially lower volumes of hazardous solvents.^24–26^

DLLME has shown good efficiency for extracting and preconcentrating hydrophobic PAHs from aqueous matrixes.^24,27^ Briefly, DLLME involves rapid injection of a small volume of an extraction solvent (immiscible with water) and dispersive solvent into the aqueous sample.^24^ Target analytes partition into the microdroplets and are subsequently recovered in a minimal volume of the organic phase after centrifugation. The large interfacial contact area between the two phases enhances mass transfer, leading to fast attainment of extraction equilibrium with high enrichment factors.^28,29^ DLLME has been applied to a wide range of matrices, including environmental samples,^25,30^ milk and dairy products,^31^ honey,^32^ and others.^33^ Optimization of DLLME conditions for targeted solutes is often performed using a one-factor-at-a-time approach, which is time-consuming, labor-intensive, and may inadequately take into account interaction between or among variables.^34^ In contrast, multivariate experimental design methodologies accommodate simultaneous variation of multiple extraction variables, facilitating identification of interaction effects and development of statistically robust models.^35–37^

Over the past decade, multivariate design of experiments (DOE) has become an important optimization strategy for process development. Compared with traditional one-factor-at-a-time approaches, DOE can reduce time, effort, and resource consumption while extracting meaningful information from a relatively small number of experimental analyses.^37–40^ Among the DOE frameworks, the Box-Behnken design (BBD), an economical variant of response surface methodology, has been widely used for process optimization, offering high statistical efficiency and good predictive performance when tuning experimental variables in analytical and industrial applications.^35,39,41–45^ This study evaluates the applicability of using RSM to optimize DLLME conditions for selective extraction and preconcentration of acenaphthene, fluorene, anthracene, phenanthrene, fluoranthene, pyrene, benzo­(a)­anthracene, and benzo­(a)­pyrene. A fractional factorial design (FrFD) was initially used to screen and identify statistically significant DLLME variables. The selected factors were then optimized using the BBD to construct quadratic models and generate response surface plots for determining optimal extraction conditions. The optimized DLLME-GC-FID method was validated, and AGREEprep and AGREE were used to quantify ‘greenness’ metric scores. Analytical practicability of the validated method was demonstrated by determining the targeted PAHs in several surface water samples.

## 2 Experimental Section

### 2.1 Chemicals and Reagents

All solvents and reagents used were of HPLC grade or analytical grade. Acenaphthene (Ace; 99%), fluorene (Flu; 98%), anthracene (Ant; ≥99%), phenanthrene (Phe; 98%), fluoranthene (Flt; 98%), pyrene (Pyr; 98%), benzo­(a)­anthracene (BaA; 99%), and benzo­(a)­pyrene (BaP; ≥96%) were obtained from Sigma-Aldrich (St. Louis, MO). HPLC-grade dichloromethane, carbon tetrachloride, chlorobenzene, cyclohexane, methanol, acetone, and acetonitrile were supplied by Classic Chemicals Sdn. Bhd. (Shah Alam, Selangor, Malaysia). Chloroform was obtained from Merck (Darmstadt, Germany). Hydrochloric acid (HCl, 37% w/w), isopropanol (99.5%), and ethanol (99.7%) were obtained from QREC (Asia) Sdn. Bhd. (Selangor, Malaysia). Sodium hydroxide (NaOH) pellets (≥98%) were obtained from Bendosen Laboratory Chemicals (Bendosen, Norway). Ultrapure water (UPW) was purified using a Milli-Q system from Millipore (Bedford, MA, USA).

### 2.2 Preparation of PAHs Stock and Standard Solution

Stock standard solutions (1000 μg/L) of each PAH were prepared in acetone. Working standard solutions were prepared by appropriate dilution of the stock solutions with acetone. All standard solutions were stored at 4 °C in the dark to minimize volatilization and photodegradation.

### 2.3 Surface Water Sample

Twenty-two surface water samples (tap water, wastewater, and lake, river, and drain water) were randomly collected from multiple states (Penang, Kedah, Negeri Sembilan, Kelantan, Selangor, and Wilayah Persekutuan Kuala Lumpur) in Malaysia. Prior to analysis, water samples were stored at 4 °C in the dark. Before analysis, samples were equilibrated to room temperature and were then centrifuged to collect the supernatant for subsequent experiments.

### 2.4 GC-FID Analysis

All experiments were performed using an Agilent 7890B GC system equipped with a G4513A autosampler and a split–splitless inlet (Agilent Technologies; Santa Clara, CA, USA). Chromatographic separation was effected by using a DB-5HT column (Agilent Technologies, Santa Clara, CA, USA; 30 m × 0.25 mm I.D. × 0.25 μm film thickness). Injection mode and oven temperature programming were optimized to achieve baseline separation of all target PAHs. Helium (99.999%) was used as the carrier gas at a constant flow rate of 1 mL/min. Splitless mode with a splitless time of 2.1 min was used, with a 1 μL injection volume. The injector temperature was maintained at 300 °C. The oven temperature program was 70 °C (hold for 3.3 min) and ramped to 300 °C (hold 5 min) at a rate of 25 °C/min. The FID temperature was set to 300 °C.

### 2.5 Design of Experiments (DOE)

#### 2.5.1 Screening Design

For multilevel FrFD, the total number of analyses required increases exponentially with the number of factors, given by the formula L^k‑p^,^38,41^ where L is the number of factor levels, k is the number of factors, and p denotes the size of the fraction of the full factorial used. A two-level FrFD is a subset of the full factorial design that evaluates factors at two settings (low and high) using 2^f‑v^ experiments^38^ (v = 1,2,3, ..., n). These designs are particularly useful to study the influence of multiple variables (extractant volume, dispersant volume, pH, centrifugation rate, and centrifugation time) on process outcomes (e.g., peak area) while reducing the number of experiments required by a full factorial design.^41^

#### 2.5.2 BBD

A three-factor rotatable BBD design was subsequently used to determine the optimal DLLME conditions. Based on the FrFD screening, the identified factors were extraction solvent volume (X_1_), dispersive solvent volume (X_2_), and sample pH (X_3_). Each factor was investigated at three levels (−1, 0, +1), corresponding to the low, medium, and high values, respectively, determined from the FrFD results. The response variable was attributed to the peak area of the PAHs determined using GC-FID. For a BBD with k factors and *C*_0_ center points, the number of experimental analyses (N) is given by^46^

$$N=2k\left(k-1\right)+C_{0}$$

The quadratic polynomial equation is generally expressed as follows

$$Y=\beta_{0}+\overset{k}{\underset{i=1}{\sum }}\beta_{i}x_{i}+\overset{k}{\underset{i=1}{\sum }}\beta_{ii}{x_{i}}^{2}+\overset{k}{\underset{i=1}{\sum }}\beta_{ij}x_{ij}$$

where β_0_ is the constant coefficient, β_*i*_ is the linear coefficient, β_*ii*_ is the coefficient of the quadratic variable, β_*ij*_ represents the interaction coefficients, and *Y* is the predicted response.^47^ ANOVA was applied to confirm the statistical significance (*p* < 0.05) of the model. The three-dimensional (3D) response surface and contour plots were used to visualize factor interactions and identify the optimum DLLME conditions.

### 2.6 The DLLME Process

The extraction method of PAHs in water samples was adopted from Pavle Jovanov^48^ with modifications. A 3.0 mL aliquot of the surface water sample was placed in a 10 mL round-bottom tube. The pH of the sample was adjusted to 7 using 1 M HCl or 1 M NaOH. Then 200 μL of acetonitrile was added as the dispersive solvent, and 100 μL of dichloromethane was added as the extraction solvent to the sample solution. The tube containing the mixture was agitated for 1 min using a vortex mixer. Subsequently, the tube underwent centrifugation at 3500 rpm for 2 min to effect two-phase separation. The sediment phase (i.e., extraction solvent) was withdrawn and transferred to a vial for GC-FID analysis.

### 2.7 Validation of the DLLME-GC-FID Method

This method was validated in terms of linear range, the limit of detection (LOD), limit of quantification (LOQ), intra- and interday precision, and recovery.^49^ Calibration curves were constructed at seven concentration levels (5, 10, 20, 30, 50, 100, and 200 μg/L). LODs were determined using signal-to-noise ratios of 3.3:1 (i.e., LOD = 3.3 × standard deviation/slope), while LOQs were estimated as LOQ = 3 × LOD, respectively. Precision was evaluated as relative standard deviation (%RSD) for retention time and peak area at three concentration levels (10, 50, and 100 μg/L), using intraday measurements (n = 9) and interday measurements (three replicates over three consecutive days, n = 27). Recovery was evaluated by spiking a surface water sample at 10, 50, and 100 μg/L. All experiments were performed in triplicate.

### 2.8 Greenness Evaluation

The Analytical Greenness Calculator (AGREE, software version 0.5 beta) and the Analytical Greenness for Sample Preparation (AGREEprep) tools were used to determine the greenness metric score of the developed methods.^50,51^ For AGREE, the 12 principles of green analytical chemistry (GAC) are converted into scores, ranging from 0 to 1, and evaluated as a weighted average to obtain the overall greenness score. For AGREEprep, the ten principles of green sample preparation are scored, with the scores from each criterion being weighted to provide an overall score ranging from 0 to 1.^51^ The results are displayed as a color-scaled pictogram.

### 2.9 Data Handling and Analysis

Data acquisition and processing were performed using Agilent MassHunter Workstation Qualitative Analysis version 10.0.10305.0 (Agilent Technologies) software. Chromatograms were generated by exporting MassHunter Workstation data into CSV file format, followed by reconstruction using Origin V.10.5.21 software (Origin Lab Corporation, Northampton, MA, USA). Minitab 19 statistical software (Minitab Inc., State College, PA, USA) was used to generate the experimental design matrix and perform statistical analysis.

## 3 Results and Discussion

The initial GC-FID conditions were adopted from Hor et al. for the determination of BAP, BAA, BBF, and chrysene in tocotrienol concentrates.^52^ Under those chromatographic conditions, baseline separation of all the targeted PAHs could not be achieved (Flt, Pyr, BaA, and BaP). Hence, GC conditions, particularly the oven temperature program, were briefly optimized to obtain baseline separation of all the targeted PAHs (Figure 1A).

**Figure 1:**
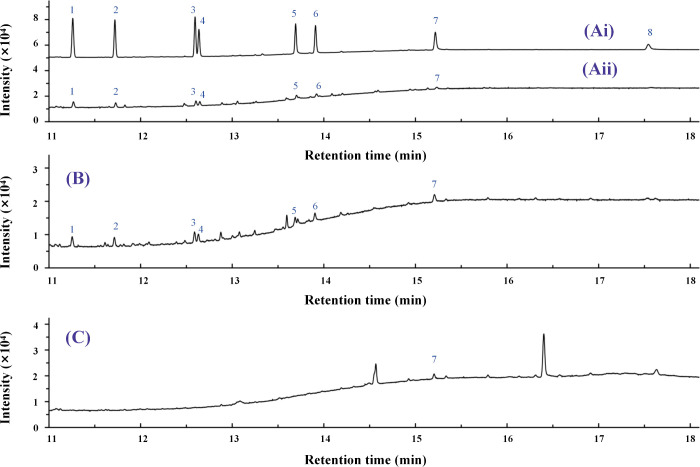
Chromatograms of (Ai) standard mixture (100 μg/L) before DLLME; (Aii) standard mixture (100 μg/L) after DLLME; (B) lake water sample spiked with PAHs (10 μg/L); and (C) wastewater sample. 1, Ace; 2, Flu; 3, Ant; 4, Phe; 5, Flt; 6, Pyr; 7, BaA; 8, BaP.

### 3.1 Evaluation of the Type of Extractants and Dispersants for DLLME

Extraction performance in DLLME is strongly influenced by the choice of extraction solvent (i.e., that which is sedimented), which controls the solutes’ partitioning behavior between the aqueous and organic phases.^24^ Five extraction solvents of different polarity, namely, dichloromethane (DCM, dielectric constant (ε): 8.93 ^53^), chloroform (ε: 4.81 ^54^), carbon tetrachloride (ε: 2.24 ^55^), chlorobenzene (ε: 5.62 ^56^), and cyclohexane (ε: 2.02 ^57^), were evaluated. Based on the overall response (i.e., peak areas), dichloromethane outperformed the other chlorinated solvents (Figure 2). This might be attributed to the stronger partitioning of PAHs into DCM, which exhibited the highest polarity among the tested extractants.^58^ Therefore, DCM was selected as the extraction solvent. It is known that the dispersive solvent serves as a “bridge” between the aqueous sample and the water-immiscible extractant, and its selection is therefore crucial to DLLME performance. Five dispersive solvents (methanol, ε: 33;^59^ ethanol, ε: 24.5;^60^ isopropanol, ε: 20.1;^61^ acetone, ε: 20.7;^62^ acetonitrile, ε: 36 ^63^) of different polarities were studied. Among these, ACN having the highest polarity provided the highest extraction response. This might be attributed to the reduced interfacial tension, which promotes formation of smaller microdroplets, and increases interfacial area for partitioning process, thereby improving extraction efficiency.^64^ Thus, DCM and ACN were selected as the extractant and dispersant, respectively, for subsequent FrFD and BBD experiments.

**Figure 2:**
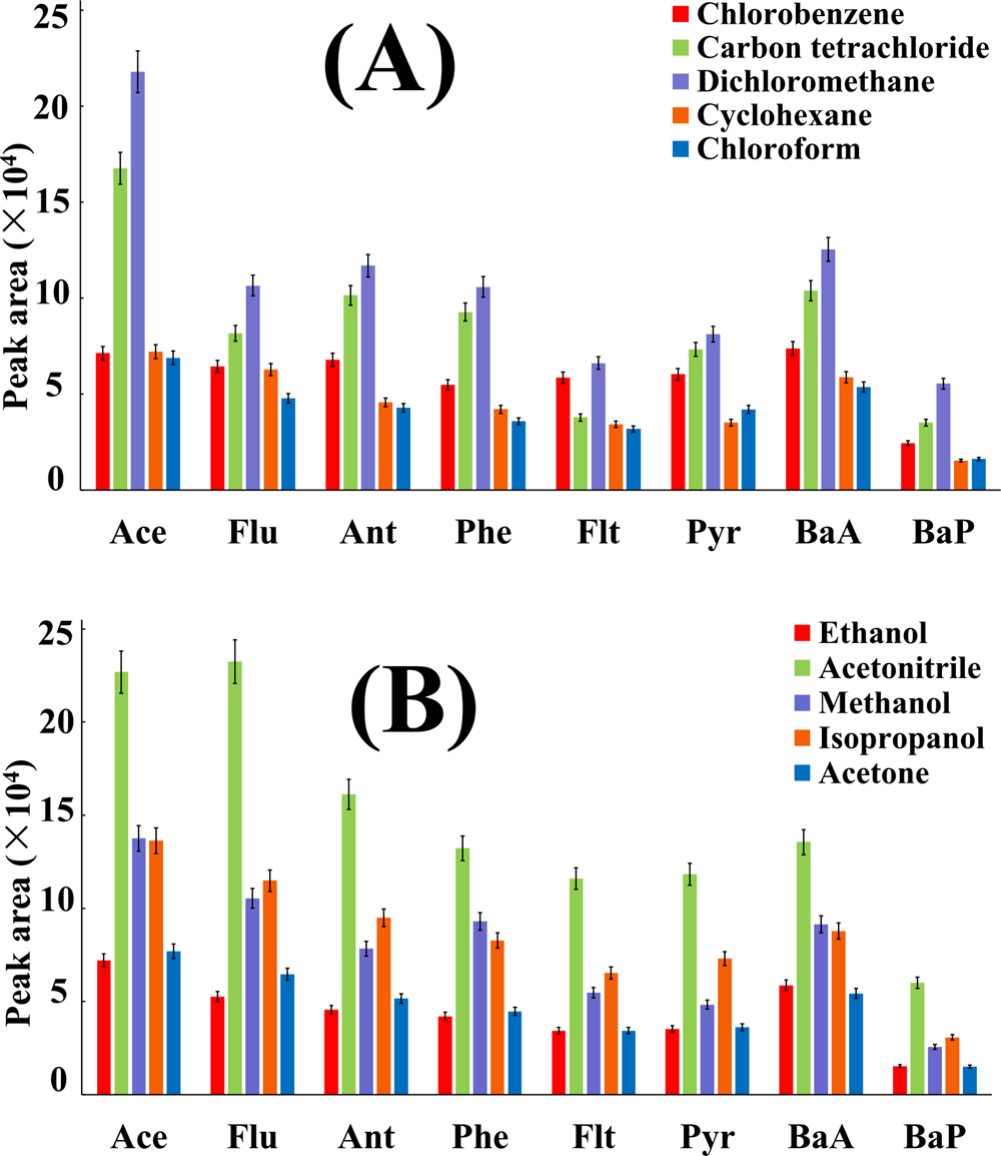
Effect of (A) selection of extraction solvent; (B) selection of dispersive solvent.

### 3.2 FrFD

FrFD was employed to screen the DLLME parameters that significantly influence extraction efficiency while reducing the experimental burden relative to a full factorial design.^41^ In this framework, the total number of experiments can be expressed as L^*k*‑*p*^, where L denotes the number of factor levels, *k* represents the number of factors, and *p* denotes the fraction of the entire full factorial design implemented. A 2^5–2^ FrFD was used to identify key variables that affect DLLME extraction efficiency and minimize the number of experiments. Five DLLME parameters, namely extractant volume (A; 200–600 μL), dispersant volume (B; 100–500 μL), pH (C; 2–12), centrifugation rate (D; 500–3500 rpm), and centrifugation time (E; 2–8 min), were evaluated. An FrFD matrix comprising 11 experiments (Table S1; Supporting Information) was devised and executed in a randomized manner to mitigate the influence of extraneous variables. The specific levels are detailed in Table 1, with each experiment conducted in triplicate.

**Table 1:** Experimental Variables and Levels of the FrFD

	Level		
Variables	Low (−1)	Central (0)	High (+1)
Extractant volume (μL)	200	400	600
Dispersant volume (μL)	100	300	500
pH	2	7	12
Centrifugal speed (rpm)	500	2000	3500
Centrifugal time (min)	2	5	8

On the basis of the Pareto plot (Figure 3), at *p* = 0.05, A (extractant volume), B (dispersant volume), C (pH), and the B*C (interaction) significantly affected the extraction efficiencies of Ace, Flu, Ant, and Flt. In contrast, D, E, and B*E did not exceed the significance threshold (*p* > 0.05), indicating no statistically significant effects for these analytes under the tested conditions. For Phe, Pyr, BaA, and BaP, A, B*C, and B*E were significant (*p* < 0.05).

**Figure 3:**
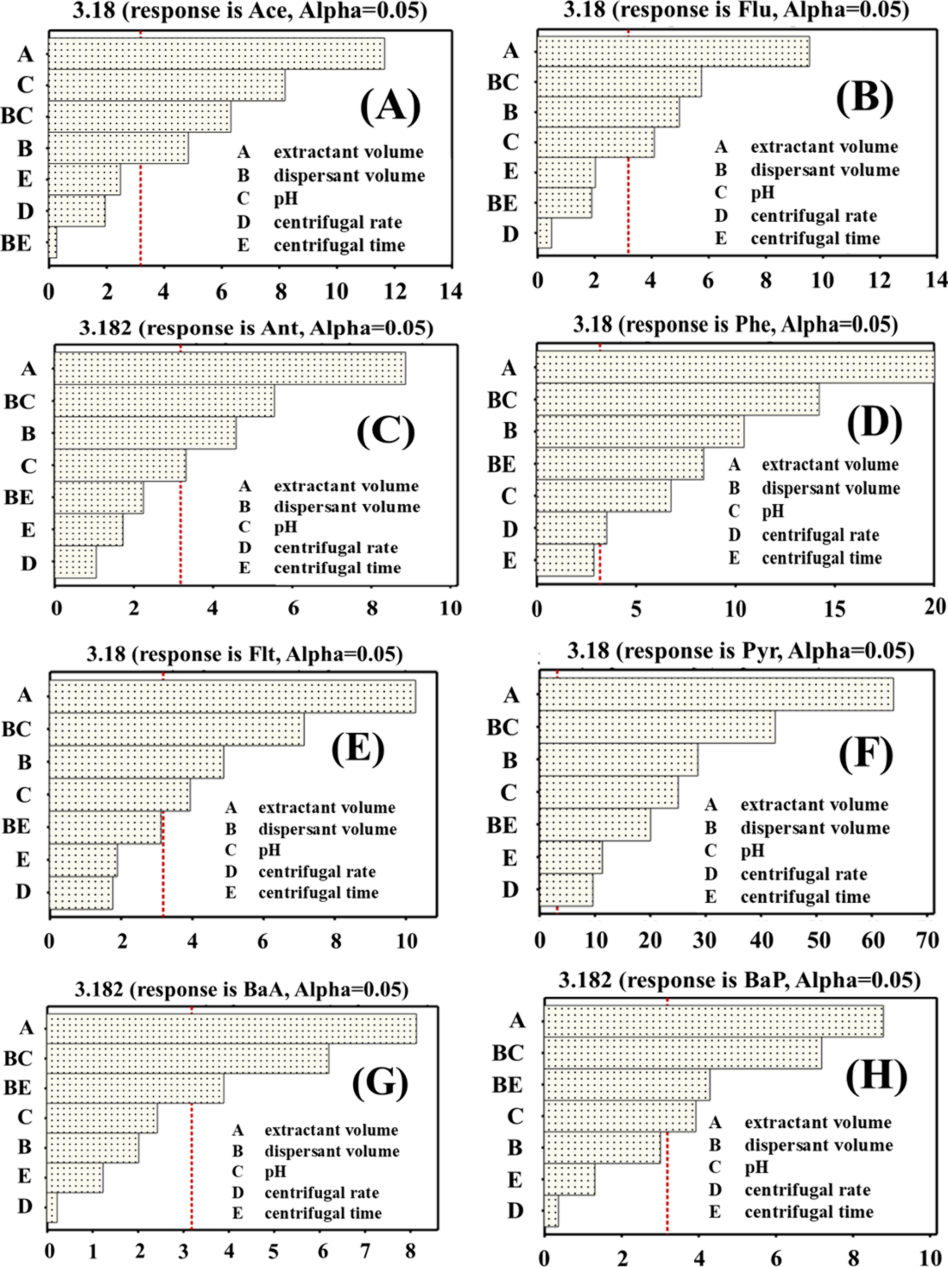
Pareto chart showing the major effects produced by the FrFD: (A) Ace; (B) Flu; (C) Ant; (D) Phe; (E) Flt; (F) Pyr; (G) BaA; (H) BaP.

Main effects plots (Figure 4Ai-Hi) and interaction plots (Figure 4Aii-Hii) further supported these findings. Specifically, A, B, C, and B*C were significant for Ace, Flu, Ant, and Flt; A, B, C, D, B*C, and B*E were significant for Phe; and A, B, C, D, E, B*C, and B*E were significant for Pyr. A, B*C, and B*E were significant for BaA; and A, C, B*C, and B*E were significant for BaP. An analysis of variance (ANOVA), supplemented by a *t*-test at the 95% confidence level, was used to evaluate the principal effects.^65^ Residual analysis (Figure S1; Supporting Information) was then used to assess model adequacy. The normal probability plot (Figure S1 Ai-Hi) showed residual points closely distributed along a straight line, supporting an approximate normal distribution. Figure S1 Aii-Hii indicated that the residual versus predicted value plots were randomly distributed around zero, indicating constant variance and an appropriate model functional form. The residual histogram (Figure S1 Aiii-Hiii) further supported the normality assumption. The residual versus observation order (Figure S1 Aiv-Hiv) showed no systematic patterns (i.e., residuals randomly distributed around zero without periodic fluctuations), indicating independence. Given that A, B, C, and B*C were consistently significant across all of the analytes in the FrFD screening, extractant volume, dispersant volume, and pH were selected as the statistically significant factors for subsequent optimization using BBD.

**Figure 4:**
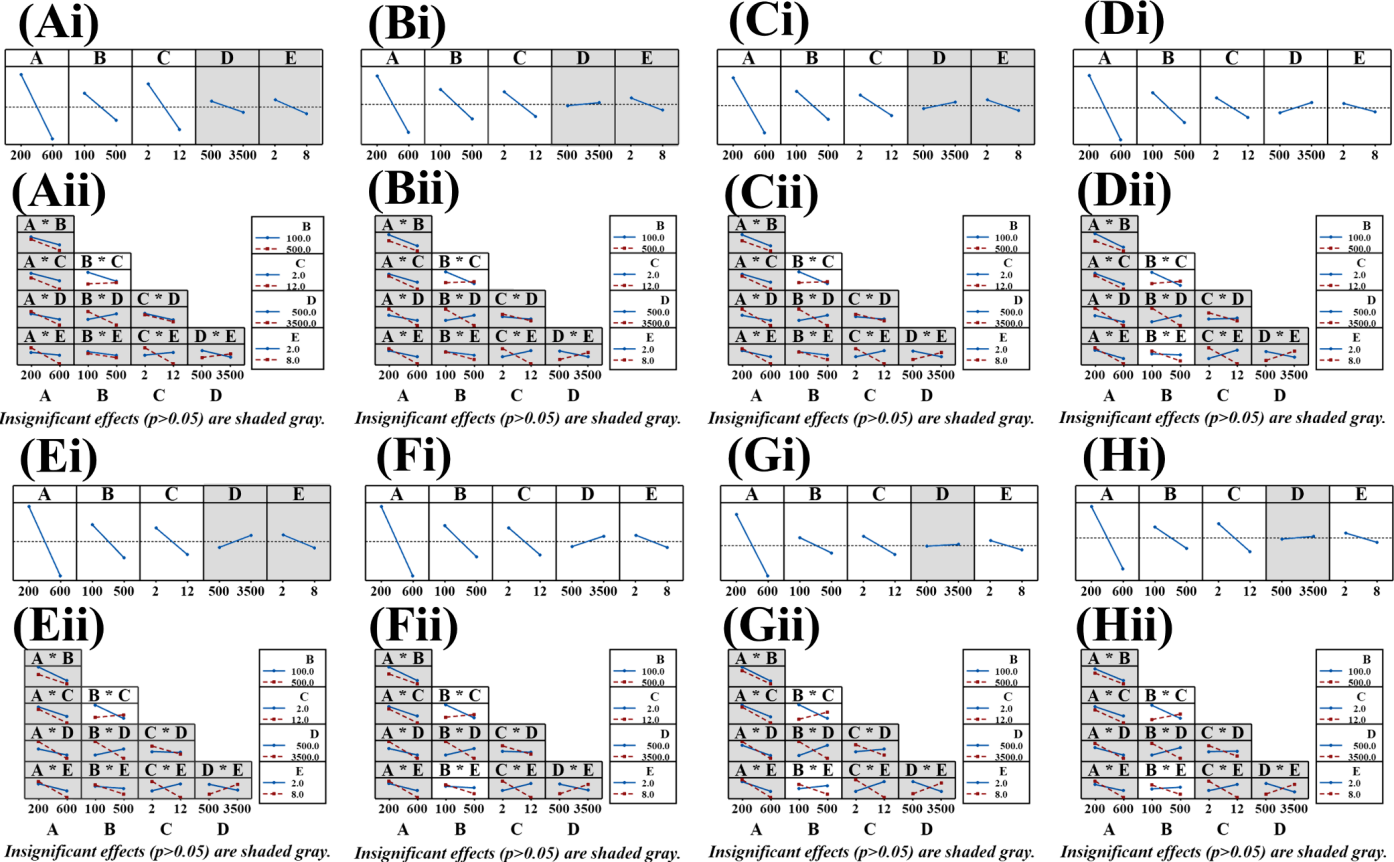
Main effects and interaction plots for PAHs obtained from FrFD: (Ai-Hi) main effects plots; (Aii-Hii) interaction plots.

### 3.3 BBD

A BBD comprising 15 experiments (each performed in triplicate), including 3 center points, was used to develop quadratic response surface models for factors A, B, and C. The BBD matrix is listed in Table S2 (Supporting Information). For each experiment, the response was defined as the geometric mean of analyte peak areas, which facilitated the visualization and comparison. Multiple linear regression analysis was applied to the responses for Ace, Flu, Ant, Phe, Flt, Pyr, BaA, and BaP to establish the relationships between response and factor levels at a 95% confidence level, as shown in Section S1 (Supporting Information).

The quality of the proposed models was evaluated using ANOVA based on Fisher’s F-test, and the results are summarized in Table 2. In the regression output, Coeff represents the estimated influence of each variable in the model, SECoeff is the standard error associated with the estimated regression coefficients, and t-statistic (Coeff/SECoeff) gauges the magnitude of the coefficient relative to the standard error. As shown in Table 2, *p* < 0.05 was considered statistically significant at the 95% confidence level. This lack of significance for the second-order polynomial model terms underscores their high predictive validity.^46^ Model adequacy was further assessed using the lack-of-fit test. A nonsignificant lack of fit (*p* > 0.05) indicates that the model describes the data satisfactorily within experimental error (Table 2). The model’s goodness of fit was also evaluated using the coefficient of determination (R^2^). A good correlation with values of R^2^ > 0.9 indicates that the model accounts for most of the response variability and is consistent with agreement between predicted and experimental values.

**Table 2:** ANOVA Regression Results for the BBD Model

Term	Mode	A^e^	B	C	A*A	B*B	C*C	A*B	A*C	B*C	Lack of Fit F-value	R^2^
Ace	27695^a^	–26563	–8158	–83	16559	2771	–4485	11165	–902	746	0.059	92.57%
	(3773)^b^	(2310)	(2310)	(2310)	(3401)	(3401)	(3401)	(3268)	(3268)	(3268)		
	(7.34)^c^	(−11.50)	(−3.53)	(−0.04)	(4.87)	(0.81)	(−1.32)	(3.42)	(−0.28)	(0.23)		
	(0.001)^d^	(0.000)	(0.017)	(0.973)	(0.005)	(0.452)	(0.244)	(0.019)	(0.794)	(0.828)		
Flu	24573	–23685	–7063	–557	14537	1896	–3229	10224	–139	830	0.056	92.77%
	(3301)	(2021)	(2021)	(2021)	(2975)	(2975)	(2975)	(2859)	(2859)	(2859)		
	(7.44)	(−11.72)	(−3.49)	(−0.28)	(4.89)	(0.64)	(−1.09)	(3.58)	(−0.05)	(0.29)		
	(0.001)	(0.000)	(0.017)	(0.794)	(0.005)	(0.552)	(0.327)	(0.016)	(0.963)	(0.783)		
Ant	23522	–22539	–6946	–648	13661	1542	–2863	9821	537	514	0.54	93.64%
	(3176)	(1945)	(1945)	(1945)	(2863)	(2863)	(2863)	(2751)	(2751)	(2751)		
	(7.41)	(−11.59)	(−3.57)	(−0.33)	(4.77)	(0.54)	(−1.00)	(3.57)	(0.20)	(0.19)		
	(0.001)	(0.000)	(0.016)	(0.753)	(0.005)	(0.613)	(0.363)	(0.016)	(0.853)	(0.859)		
Phe	17167	–15728	–4518	–18	9312	986	–295	5101	–394	541	0.058	97.93%
	(1131)	(693)	(693)	(693)	(1019)	(1019)	(1019)	(979)	(979)	(979)		
	(15.18)	(−22.71)	(−6.52)	(−0.03)	(9.14)	(0.97)	(−0.29)	(5.21)	(−0.40)	(0.55)		
	(0.000)	(0.000)	(0.001)	(0.980)	(0.000)	(0.378)	(0.784)	(0.003)	(0.704)	(0.605)		
Flt	16367	–17200	–5170	–374	10695	1303	–1993	7453	112	571	0.052	92.71%
	(2411)	(1476)	(1476)	(1476)	(2173)	(2173)	(2173)	(2088)	(2088)	(2088)		
	(6.79)	(−11.65)	(−3.5)	(−0.25)	(4.92)	(0.60)	(−0.92)	(3.57)	(0.05)	(0.27)		
	(0.001)	(0.000)	(0.017)	(0.810)	(0.004)	(0.575)	(0.401)	(0.016)	(0.959)	(0.795)		
Pyr	18100	–17356	–5060	–128	10488	837	–2015	7426	27	341	0.055	94.01%
	(2184)	(1338)	(1338)	(1338)	(1969)	(1969)	(1969)	(1892)	(1892)	(1892)		
	(8.29)	(−12.97)	(−3.78)	(−0.10)	(5.33)	(0.42)	(−1.02)	(3.93)	(0.01)	(0.18)		
	(0.000)	(0.000)	(0.013)	(0.928)	(0.003)	(0.689)	(0.353)	(0.011)	(0.989)	(0.864)		
BaA	13783	–13775	–3731	–403	8408	134	–935	4998	389	347	0.056	96.26%
	(1344)	(823)	(823)	(823)	(1211)	(1211)	(1211)	(1164)	(1164)	(1164)		
	(10.26)	(−16.74)	(−4.53)	(−0.49)	(6.94)	(0.11)	(−0.77)	(4.29)	(0.33)	(0.30)		
	(0.000)	(0.000)	(0.006)	(0.645)	(0.001)	(0.916)	(0.475)	(0.008)	(0.752)	(0.777)		
BaP	6101	–5930	–1351	107	3455	–148	46	1532	–381	82	0.632	98.23%
	(386)	(236)	(236)	(236)	(348)	(348)	(348)	(334)	(334)	(334)		
	(15.81)	(−25.10)	(−5.72)	(0.45)	(9.93)	(−0.42)	(0.13)	(4.58)	(−1.14)	(0.25)		
	(0.000)	(0.000)	(0.002)	(0.671)	(0.000)	(0.689)	(0.899)	(0.006)	(0.306)	(0.815)		

a Coeff.

b SECoeff.

c T (Coeff/SECoeff)

d p-Value (P).

e A, extractant volume, B, dispersant volume, C, pH.

Figure 5 shows residual diagnostics for the BBD-fitted model to evaluate model adequacy. In the normal probability plots (Figure 5Ai-Hi), residual points are distributed approximately along a straight line, indicating that the normality assumption is valid. In the residuals versus predicted values plots (Figure 5Aii-Hii), residuals are randomly dispersed around zero, supporting homogeneity of variance and an appropriate model functional form. The residual histogram (Figure 5Aiii-Hiii) shows approximately symmetrical distributions, supporting the statistical hypothesis that residuals follow a normal distribution. In the residuals versus observation order plots (Figure 5Aiv-Hiv), residuals scattered randomly around the zero reference line without systematic fluctuations or patterns, suggesting that the residuals are independent. Figure S2 (Supporting Information) shows the residuals plotted against factors plots. The residuals remain randomly distributed around zero and do not exhibit systematic increases or decreases in trend with factor magnitude, indicating that the model adequately captures the underlying relationships with no nonlinear or interaction terms having been omitted. All the obtained diagnostic results indicate the model assumptions are reasonably satisfied and that the overall fit is adequate.

**Figure 5:**
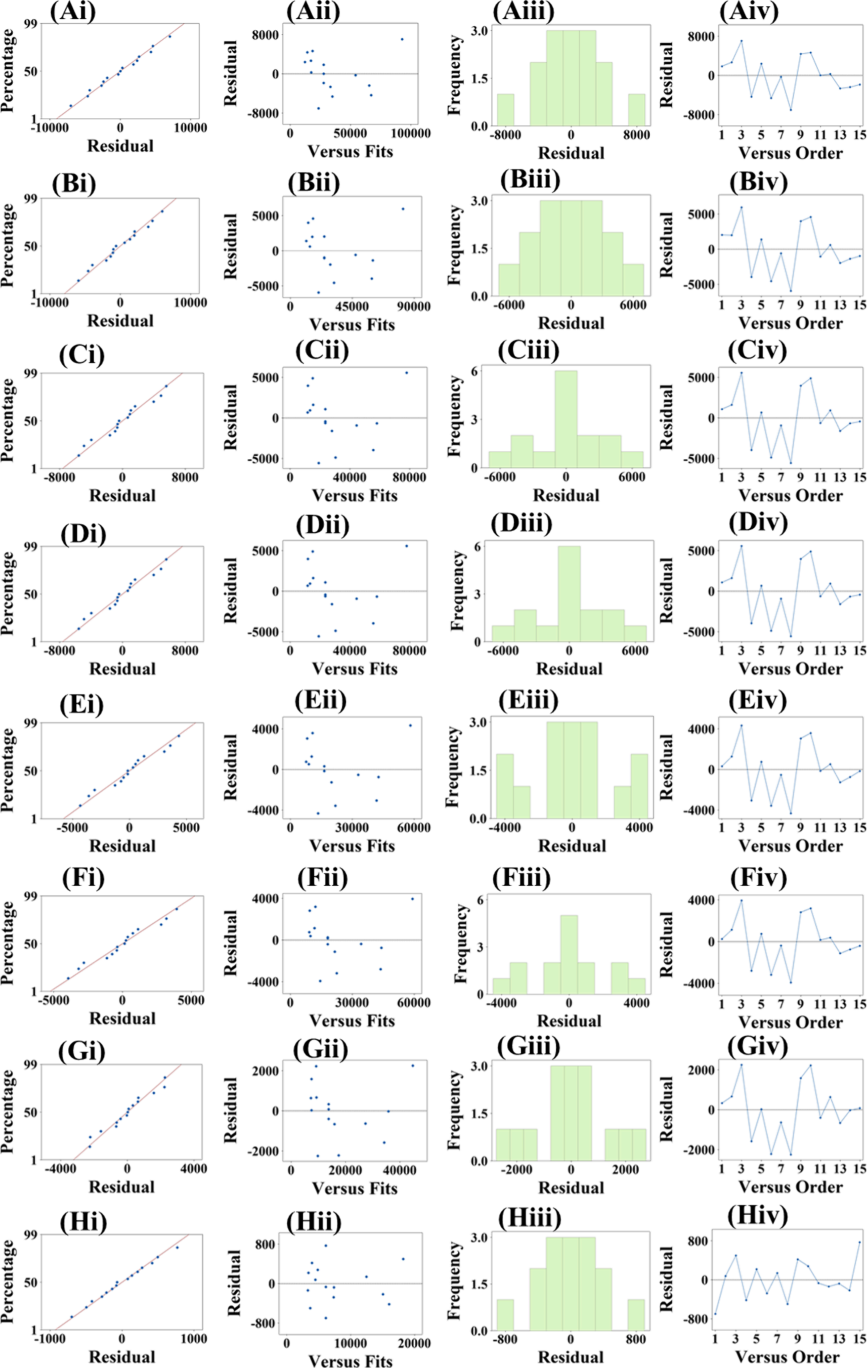
Residual analysis plots for BBD models: (Ai-Hi) normal probability plots; (Aii-Hii) residual versus predicted value plots; (Aiii-Hiii) residual histogram; (Aiv-Hiv) residual versus observation order.

Three-dimensional response surface plots (Figure 6) were constructed to visualize the effect of the variables on response intensity of PAHs. At pH 7 (Figure 6Ai-Hi), the effects of extraction volume (200–600 μL) and dispersant volume (100–500 μL) were evaluated simultaneously. Increasing the DCM volume is expected to increase the volume of the sedimented organic phase after centrifugation, which can reduce PAH response through dilution. In contrast, the volume of ACN used affects formation of the water/acetonitrile/dichloromethane system and the extent to which the extractant is in the aqueous phase.^48^ As shown in Figure 6Aii-Hii, at a dispersant volume of 100 μL, PAH response intensity decreased as the DCM (extractant) volume increased. The observed trend may be attributed to increasing PAH solubility in water with a higher ACN content and reducing partitioning into the extractant phase, thereby lowering extraction efficiency.^64^ As illustrated in Figure 6Aii-Hii and Figure 6Aiii-Hiii, the response intensity increased as the pH of the sample solution shifted from acidic to near-neutral conditions and then decreased under alkaline conditions. The observed behavior is in agreement with prior findings that PAHs are largely nonpolar and lack ionizable functional groups (e.g., −COOH, −OH, −NH_2_), and thus, extraction efficiency is typically higher near neutral pH.^66,67^ Using the established model, optimal DLLME conditions were determined by numerical optimization of the response surface: extractant volume 200 μL, dispersant volume 100 μL, and pH 6.91. Under these conditions, the predicted extraction efficiency was 90.1%. For practicality, the optimized pH was rounded to 7.0, and the conditions of 200 μL of DCM, 100 μL of ACN, and pH 7 were used for subsequent experiments. Figure 1A shows the GC-FID chromatograms of PAHs (100 ppb) before and after DLLME. Application of the optimized DLLME conditions provided enrichment factors of 19.6–48.1 for the studied PAHs.

**Figure 6:**
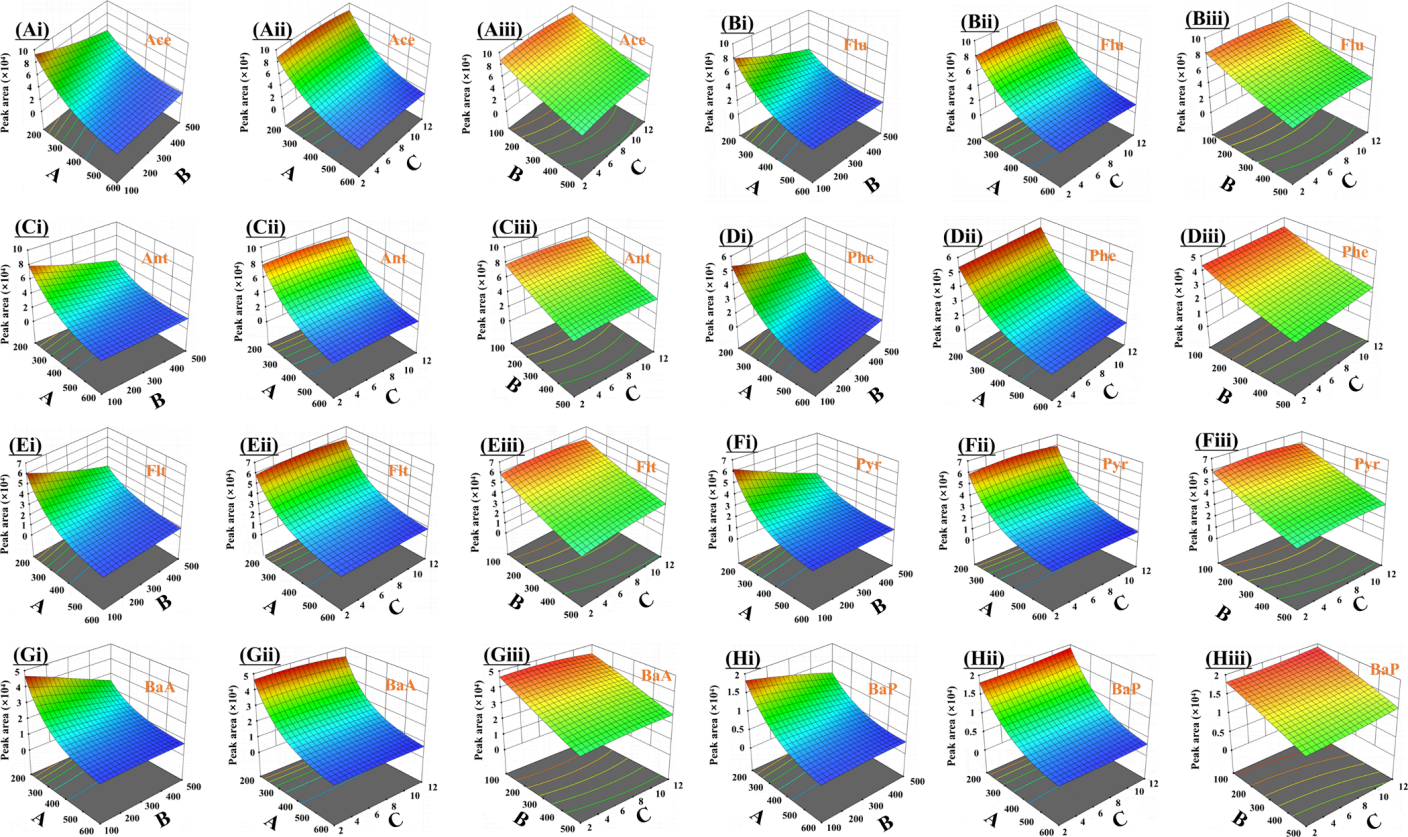
Three-dimensional response surface plots illustrating the effects of DLLME variables on peak area: (Ai-Hi) extractant volume versus dispersant volume; (Aii-Hii) extractant volume versus pH; (Aiii-Hiii) dispersive solvent volume versus pH, (A) extractant volume; (B) dispersant volume; (C) pH.

### 3.4 Method Validation

Validation of the optimized DLLME-GC-FID method (Table 3) was conducted with reference to the ICH guidelines.^49^ Good linearity was achieved across the studied range (5–200 μg/L), with correlation coefficients ≥ 0.99. The limit of detections (LODs) ranged from 0.11 to 0.33 μg/L, which are substantially lower than those reported for μSPE-GC-FID (8.47–27.34 μg/L),^68^ dynamic LPME-HPLC-UV (0.45–0.60 μg/L),^69^ and headspace solvent microextraction-GC-FID (7–41 μg/L),^70^ and are comparable with the use of polyethylene terephthalate membrane modified with cobalt zeolitic imidazolate framework (PET/Co ZIF)-HPLC-UV (0.05–0.34 μg/L) methods.^71^ Intraday precision was evaluated using three different independent preparations at 10, 50, and 100 μg/L analyzed on the same day (n = 9), while interday precision was assessed at the same concentrations over three consecutive days (n = 27). Retention time RSDs were ≤ 0.10%, and peak area RSDs ranged from 0.16% to 9.51%, which is better than the reported GC × GC/Q-TOFMS approach (RSD 11–17%) for analysis of PAHs in water.^72^ A representative GC-FID chromatogram of a spiked water sample (10 μg/L) is illustrated in Figure 1(B). The recoveries obtained at three spiking concentration levels (10, 50, and 100 μg/L) ranged from 65.1 ± 1.4% to 110.8 ± 4.5%. These values are comparable to those reported for SPE-LC-FLD (91 ± 6% to 160 ± 9%)^21^ and headspace solid-phase microextraction-GC-FID (67.87 ± 1.6% to 98.74 ± 3.3%).^73^

**Table 3:** Linearity, Limits of Detection and Quantification, Accuracy, and Intra- and Interday Precision for the Target PAHs Determined Using the Developed DLLME-GC-FID Method

							Mean recovery (% ± SD)			Intraday precision (*n* = 9) (%RSD)			Interday precision (*n* = 27) (%RSD)		
PAH	Linear Range (μg/L)	Regression equation	R^2^	LOD (μg/L)	LOQ (μg/L)	Enrichment factor	10 (μg/L)	50 (μg/L)	100 (μg/L)	10 (μg/L)	50 (μg/L)	100 (μg/L)	10 (μg/L)	50 (μg/L)	100 (μg/L)
Ace	5–200	y = 824.46x + 4736	0.9939	0.13	0.39	22.03	78.64 ± 7.36	87.04 ± 3.73	93.93 ± 0.85	3.30	6.36	4.36	6.84	2.40	4.64
										(0.01)	(0.01)	(0.02)	(0.01)	(0.02)	(0.01)
Flu	5–200	y = 834.12x – 628.22	0.9983	0.11	0.34	27.55	77.70 ± 5.91	91.51 ± 2.93	84.32 ± 3.54	2.62	2.72	0.16	8.47	7.79	4.92
										(0.01)	(0.01)	(0.01)	(0.02)	(0.01)	(0.01)
Ant	5–200	y = 807.2x + 39.684	0.9996	0.22	0.65	25.97	72.49 ± 1.11	90.79 ± 2.52	81.24 ± 3.72	3.09	6.98	2.45	8.41	7.53	6.38
										(0.01)	(0.01)	(0.01)	(0.01)	(0.02)	(0.01)
Phe	5–200	y = 526.56x + 1200.2	0.9974	0.25	0.76	19.63	65.05 ± 1.40	102.55 ± 7.01	97.23 ± 1.90	5.19	5.10	2.49	9.51	5.42	6.71
										(0.01)	(0.01)	(0.01)	(0.01)	(0.01)	(0.01)
Flt	5–200	y = 584.53x – 2060.2	0.9984	0.22	0.66	48.12	96.72 ± 5.32	107.97 ± 0.51	90.82 ± 4.65	0.99	5.24	2.19	8.81	9.16	5.76
										(0.01)	(0.01)	(0.01)	(0.01)	(0.01)	(0.01)
Pyr	5–200	y = 609.35x – 77.17	0.9995	0.12	0.36	29.07	74.58 ± 1.46	98.69 ± 2.36	89.84 ± 3.88	2.71	2.63	1.55	8.87	4.90	2.54
										(0.01)	(0)	(0.01)	(0.01)	(0.01)	(0.01)
BaA	5–200	y = 491.95x – 314.27	0.9973	0.25	0.75	31.03	82.53 ± 3.81	110.76 ± 4.52	97.37 ± 4.07	4.55	2.46	1.97	8.44	2.83	6.83
										(0.01)	(0.01)	(0.01)	(0.01)	(0.01)	(0.01)
BaP	5–200	y = 204.44x + 408.01	0.9968	0.33	0.98	34.16	77.46 ± 3.13	97.11 ± 1.81	91.78 ± 2.39	5.26	3.75	1.72	4.52	5.57	4.50
										(0.01)	(0.02)	(0.01)	(0.10)	(0.03)	(0.01)

### 3.5 Method Greenness Assessment

AGREE and AGREEprep were applied to evaluate the extent to which the proposed method aligns with the principles of green analytical chemistry. These tools generate a color-coded pictogram diagram and an overall greenness score (0–1), which serves as an index of method sustainability and environmental compatibility.^50,51^ The greenness profile and performance of the proposed method for PAHs determination in surface water were evaluated against other reported approaches. As shown in Figure 7(A), SPME,^74^ μ-SPE,^68^ dynamic-LPME,^69^ and LPE^75^ methods achieved AGREEprep scores of 0.65, 0.53, 0.44 and 0.33, respectively, whereas DLLME yielded a score of 0.59. The lower scores for dynamic-LPME and LPE were attributed to the greater reagent consumption, longer processing time, and operational complexity. In particular, the LPE procedure involves multiple steps and the use of relatively large quantities of chromic acid, rendering the workflow more laborious than dynamic-LPME and DLLME.^69,75^ By contrast, the higher score for SPME was attributed to its low sample requirement (100 μL) and minimal solvent use.^74^ Figure 7(B) shows that the overall AGREE score for the developed DLLME-GC-FID method (0.57) exceeded that of the dynamic LPME-HPLC-UV method (0.34)^69^ and LPE-GC-MS method (0.24), which is consistent with the DLLME workflow, that demonstrated fewer procedural steps, and reduced waste generation. Although the vacuum-assisted total-vaporization SPME-GC-FID method (0.63)^74^ and μ-SPE-GC-FID method (0.52)^68^ achieved relatively high scores due to smaller sample inputs, the DLLME-GC-FID method offers a simpler operational workflow and a shorter extraction time and chromatographic run time.

**Figure 7:**
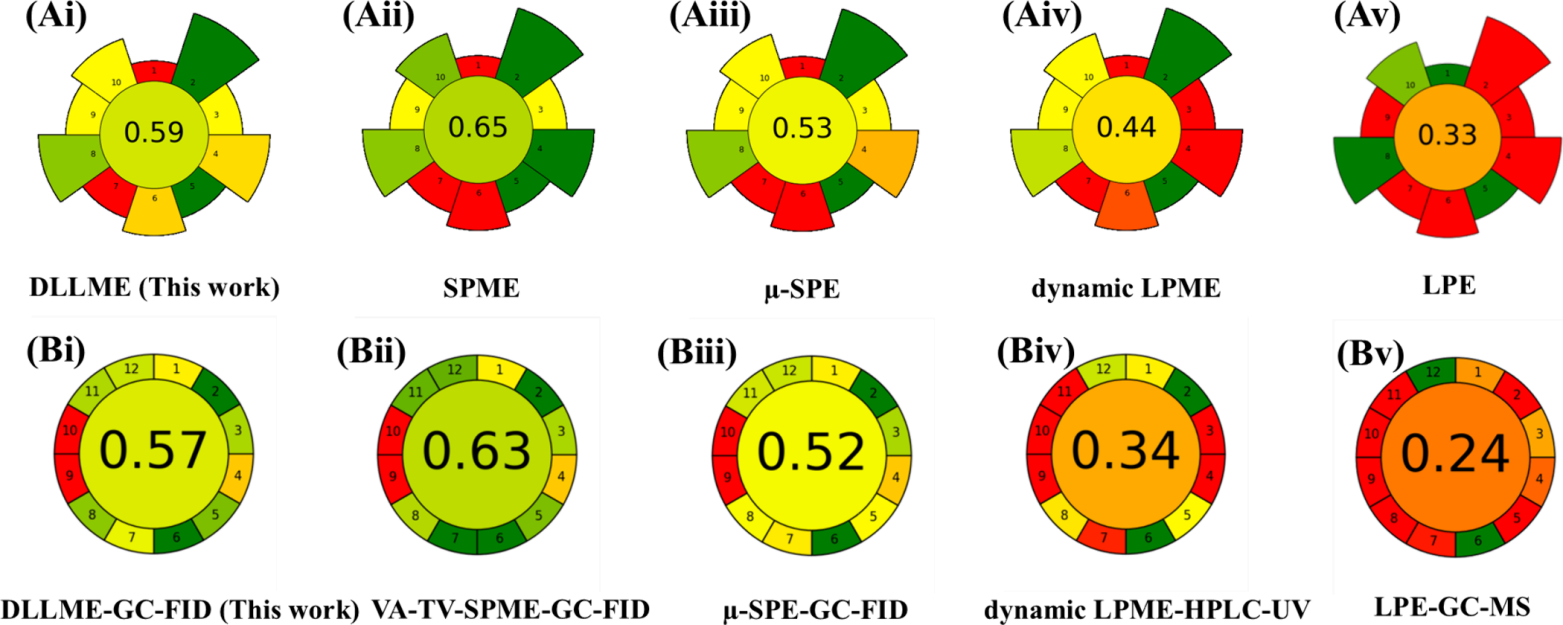
Pictograms obtained from AGREEprep and AGREE assessment of procedures for PAH determination in surface water: (A) AGREEprep scores for (i) DLLME (this work), (ii) SPME, (iii) μ-SPE, (iv) dynamic LPME, (v) LPE; (B) AGREE scores for (i) DLLME-GC-FID (this work), (ii) VA-TV-SPME-GC-FID, (iii) μ-SPE-GC-FID, (iv) dynamic LPME-HPLC-UV, (v) LPE-GC-MS.

### 3.6 DLLME-GC-FID Analysis of Surface Water Samples

To further evaluate the practical applicability of the developed approach, the method was applied to the determination of PAHs in 22 surface water samples (tap, river, lake, drain water, and wastewater), as summarized in Table S3 (Supporting Information). Total PAH concentrations in tap water samples ranged from 0.80 to 1.27 μg/L, while drain water samples contained 0.91 to 1.00 μg/L. River and lake water samples showed higher total PAH concentrations (0.9–2.01 μg/L). The highest concentrations were observed in wastewater, ranging from 1.73 to 4.05 μg/L. The EU has established a limit of 0.1 μg/L for total PAHs in drinking water.^12^ In heavily polluted waterways, PAH concentrations up to 10 μg/mL have been reported.^13^ Across the 22 samples analyzed, BaA was detected in nearly all samples, with a mean concentration of 1.62 μg/L, exceeding the EU drinking water limit. Comparable BaA levels (0.40 to 4.7 μg/L) have been reported in seawater around Langkawi Island.^76^ In contrast, reported BaA concentrations in Nigeria Allor River water were substantially lower (0.3–2.8 ng/L), while surface sediments from the same river contained 9.6–19.3 μg/kg BaA.^77^ The occurrence of BaA in drain water might be attributed to atmospheric deposition, where PAHs formed during combustion process can be scavenged by rainfall and subsequently transported into drainage networks.^21^ Additionally, the sorption of BaA to suspended particulates and organic matter, followed by accumulation in sediments, subsequent leaching, and sediment-water exchange, can reintroduce BaA into the overlying water column or systems.^76,78^ These findings highlight the need for continued monitoring given their occurrence and potential health risks.

## 4 Conclusion

An improved and environmentally friendly DLLME-GC-FID method was described for the quantitative analysis of PAHs in surface water. RSM was applied by first screening influential variables using a fractional factorial design, followed by the Box-Behnken design optimization of the DLLME parameters. In contrast to single-factor experimentation, the fitted model and 3D response surface plots showed factor interactions and their combined effects on extraction performance, facilitating near-optimal conditions to be identified with a limited number of experiments. Using the optimized conditions, low detection limits, good enrichment factors, acceptable reproducibility, and recoveries were achieved. The validated analytical features indicate that the proposed method is robust and cost-effective for the routine screening of PAHs in terrestrial water bodies.

## Supplementary Material



## Notes

Table S1 Experimental variables evaluated during method optimization. Table S2 BBD matrix used for optimization of the experimental variables. Table S3 Concentrations (ng/mL) of target PAHs detected in surface water samples. Section S1: Multiple linear regression applied to the responses for Ace, Flu, Ant, Phe, Flt, Pyr, BaA, and BaP to model the relationships between the response variables and factor levels at the 95% confidence level. Figure S1 Residual analysis plots of PAHs in FrFD: (Ai-Hi) normal probability plots; (Aii-Hii) residual versus predicted value plots; (Aiii-Hiii) residual histogram; (Aiv-Hiv) residual versus observation order. Figure S2 Residual versus vs factors plots: (A) Ace; (B) Flu; (C) Ant; (D) Phe; (E) Flt; (F) Pyr; (G) BaA; (H) BaP (PDF)
