# Supplementary material for: Experimental Design-Based Dispersive Liquid–Liquid Microextraction with GC-FID for Determination of Polycyclic Aromatic Hydrocarbons in Surface Water
Source: ACS Omega. 2026 May 19;11(21):31418–31. doi: 10.1021/acsomega.6c01489 (PMC13234638; doi:10.1021/acsomega.6c01489)
Supplement: Supplementary file 1 [file ao6c01489_si_001.pdf]

Experimental design-based dispersive liquid-liquid microextraction with GC-FID for determination of polycyclic aromatic hydrocarbons in surface water

Chen Minghong[a,1], Song Peiyu[a,1], Tien Ping Lee[b], Garret Chan Zhe Ming[a], Philip J. Marriott[c], Yong Foo Wong[a,*]

[a] Center for Research on Multidimensional Separation Science, School of Chemical Sciences, Universiti Sains Malaysia, 11800 USM, Penang, Malaysia
[b] The Royal College of Surgeons in Ireland and University College Dublin Malaysia Campus, 4 Jalan Sepoy Lines, George Town 10450, Penang, Malaysia
[c] Australian Centre for Research on Separation Science, School of Chemistry, Monash University, Wellington Road, Clayton, Victoria 3800, Australia

Submission to

ACS OMEGA

[1], the authors contributed equally to the work.

* Corresponding author
Tel: +604 653 4031
Fax: +604 657 4854
E-mail: wongyongfoo@usm.my (Y.F. Wong)

Table S1 Experimental variables evaluated during method optimization.

Run sequence	Extractant volume	Dispersant volume	pH	Centrifugation speed	Centrifugation time
1	600	100	12	500	8
2	400	300	7	2000	5
3	600	500	2	3500	2
4	200	100	2	3500	8
5	200	500	12	500	2
6	400	300	7	2000	5
7	200	100	12	3500	2
8	600	100	2	500	2
9	600	500	12	3500	8
10	200	500	2	500	8
11	400	300	7	2000	5

Table S2 BBD matrix used for optimization of the experimental variables.

Run sequence	Extractant volume(μL)	Dispersant volume(μL)	pH
1	400	300	7
2	400	500	2
3	200	100	7
4	200	300	12
5	600	300	12
6	400	100	2
7	200	500	7
8	600	500	7
9	600	300	2
10	400	500	12
11	400	300	7
12	600	100	7
13	400	100	12
14	200	300	2
15	400	300	7

Table S3 Concentrations (ng/mL) of target PAHs detected in surface water samples.

Surface water samples	Average concentration ± SD							
	Ace	Flu	Ant	Phe	Flt	Pyr	BaA	BaP
tap water								
1	ND	ND	ND	ND	ND	ND	0.80±0.04	ND
2	ND	ND	BLD	ND	ND	ND	1.07±0.02	ND
3	ND	ND	ND	ND	ND	ND	1.27±0.08	ND
4	ND	ND	BLD	ND	ND	ND	0.99±0.13	ND
5	ND	ND	ND	ND	ND	ND	1.16±0.08	ND
Wastewater								
1	ND	ND	ND	ND	ND	ND	2.22±0.02	ND
2	ND	ND	ND	ND	ND	ND	4.05±0.09	ND
3	ND	ND	ND	ND	ND	ND	1.73±0.13	ND
4	ND	ND	ND	ND	ND	ND	2.38±0.05	ND
5	ND	1.15±0.01	0.20±0.04	ND	ND	ND	1.76±0.04	ND
Lake								
1	ND	ND	BLD	ND	ND	ND	0.99±0.25	ND
2	ND	ND	ND	ND	ND	ND	2.01±0.21	ND
River								
1	ND	ND	BLD	ND	ND	ND	2.16±0.07	ND
2	ND	ND	BLD	ND	ND	ND	2.26±0.11	ND
3	ND	ND	ND	ND	ND	ND	ND	ND
4	ND	ND	ND	ND	ND	ND	1.11±0.19	ND
5	ND	ND	ND	ND	ND	ND	1.78±0.26	ND
6	ND	ND	BLD	ND	ND	ND	0.92±0.19	ND
7	ND	ND	BLD	ND	ND	ND	1.99±0.02	ND
8	ND	ND	BLD	ND	ND	ND	1.45±0.06	ND
drain water								
1	ND	ND	ND	ND	ND	ND	0.91±0.01	ND
2	ND	ND	BLD	ND	ND	ND	1.00±0.08	ND

N: total number of water samples analyzed;
BLD, below the limit of detection;
ND, not detected.
Ace, acenaphthene; Flu, fluorene; Ant, anthracene; Phe, phenanthrene; Flt, fluoranthene; Pyr, pyrene; BaA, benzo(a)anthracene; and BaP, benzo(a)pyrene.

Section S1: Multiple linear regression applied to the responses for Ace, Flu, Ant, Phe, Flt, Pyr, BaA, and BaP to model the relationships between the response variables and factor levels at the 95% confidence level.

Y(Ace)=189393-541.4 extractant volume-199.2 dispersant volume +2632 pH+ 0.4140 extractant volume*extractant volume+0.0693 dispersant volume* dispersant volume-179 pH*pH+0.2791 extractant volume*dispersant volume -0.90 extractant volume*pH+0.75 dispersive volume*pH

Y(Flu)=171426-484.9 extractant volume-171.8 dispersant volume+1503 pH+ 0.3634 extractant volume*extractant volume+0.0474 dispersant volume* dispersant volume-129 pH*pH+0.2556 extractant volume*dispersant volume -0.14 extractant volume*pH+0.83 dispersive volume*pH

Y(Ant)=164475-463.3 extractant volume-159.7 dispersant volume +1105 pH+ 0.3415 extractant volume*extractant volume+0.0385 dispersant volume* dispersant volume-115 pH*pH+0.2455 extractant volume*dispersant volume +0.54 extractant volume*pH+0.51 dispersive volume*pH

Y(Phe)=109646-300.4 extractant volume-92.2 dispersant volume+157 pH+ 0.2328 extractant volume*extractant volume+0.0246 dispersant volume* dispersant volume-11.8 pH*pH+0.1275 extractant volume*dispersant volume -0.394 extractant volume*pH+0.541 dispersive volume*pH

Y(Flt)=124726-356.6 extractant volume-123.9 dispersant volume+825 pH+ 0.2674 extractant volume*extractant volume+0.0326 dispersant volume* dispersant volume-79.7pH*pH+0.1863 extractant volume*dispersant volume +0.11 extractant volume*pH+0.57 dispersive volume*pH

Y(Pyr)=123537-352.4 extractant volume-114.5 dispersant volume+990 pH+ 0.2622 extractant volume*extractant volume+0.0209 dispersant volume* dispersant volume-80.6 pH*pH+0.1857 extractant volume*dispersant volume +0.03 extractant volume*pH+0.34 dispersive volume*pH

Y(BaA)=96404-277.2 extractant volume-73.1 dispersant volume+183 pH+ 0.2102 extractant volume*extractant volume+0.0033 dispersant volume* dispersant volume-37.4 pH*pH+0.1250 extractant volume*dispersant volume +0.39 extractant volume*pH+0.35 dispersive volume*pH

Y(BaP)=37118-107.57 extractant volume-20.44 dispersant volume+123 pH+ 0.08637 extractant volume*extractant volume-0.00369 dispersant volume* dispersant volume+1.8 pH*pH+0.03830 extractant volume*dispersant volume

-0.381 extractant volume*pH+0.082 dispersive volume*pH

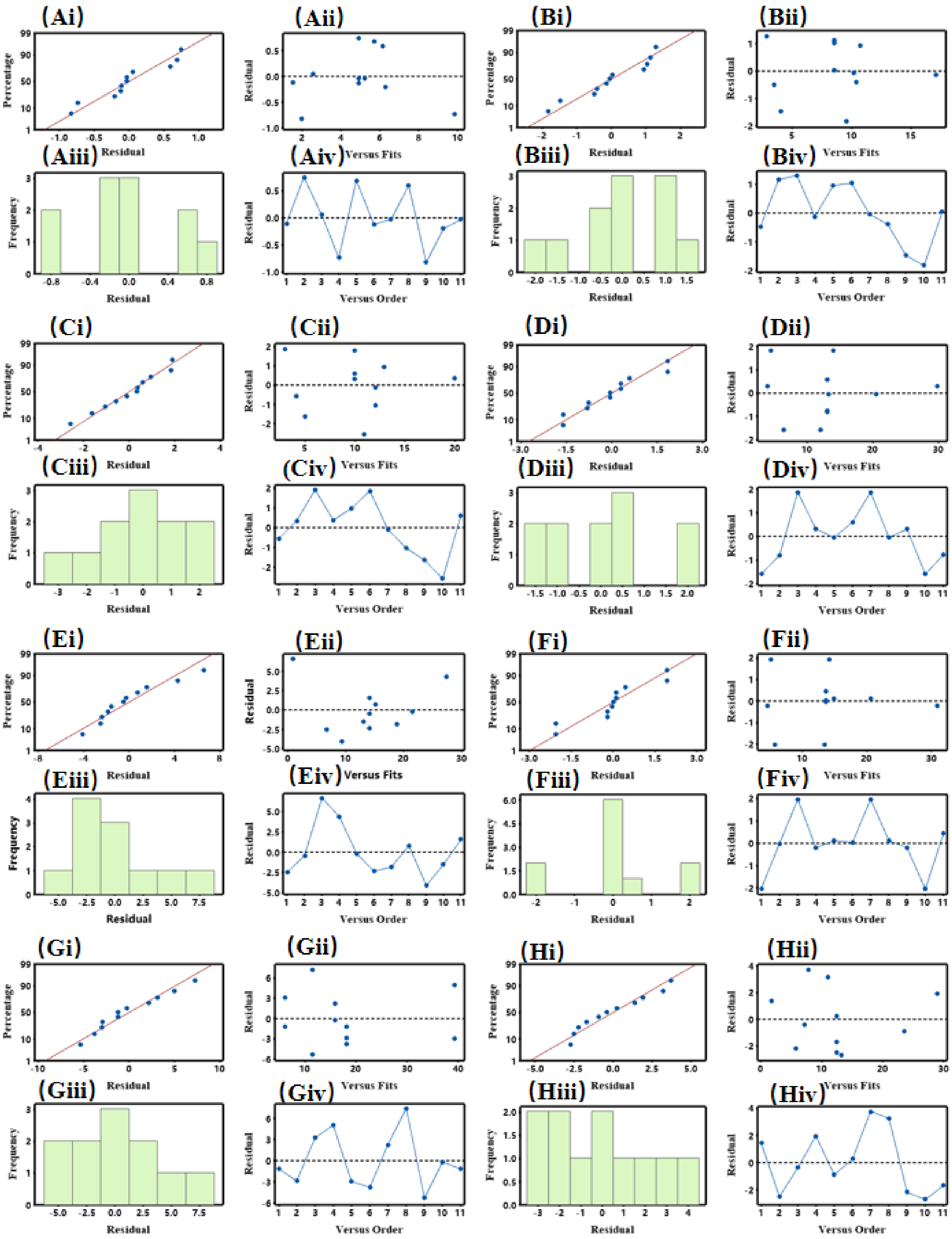

Fig. S1 Residual analysis plots of PAHs in FrFD: (Ai-Hi) normal probability plots; (Aii-Hii) residual versus predicted value plots; (Aiii-Hiii) residual histogram; (Aiv-Hiv) residual versus observation order.

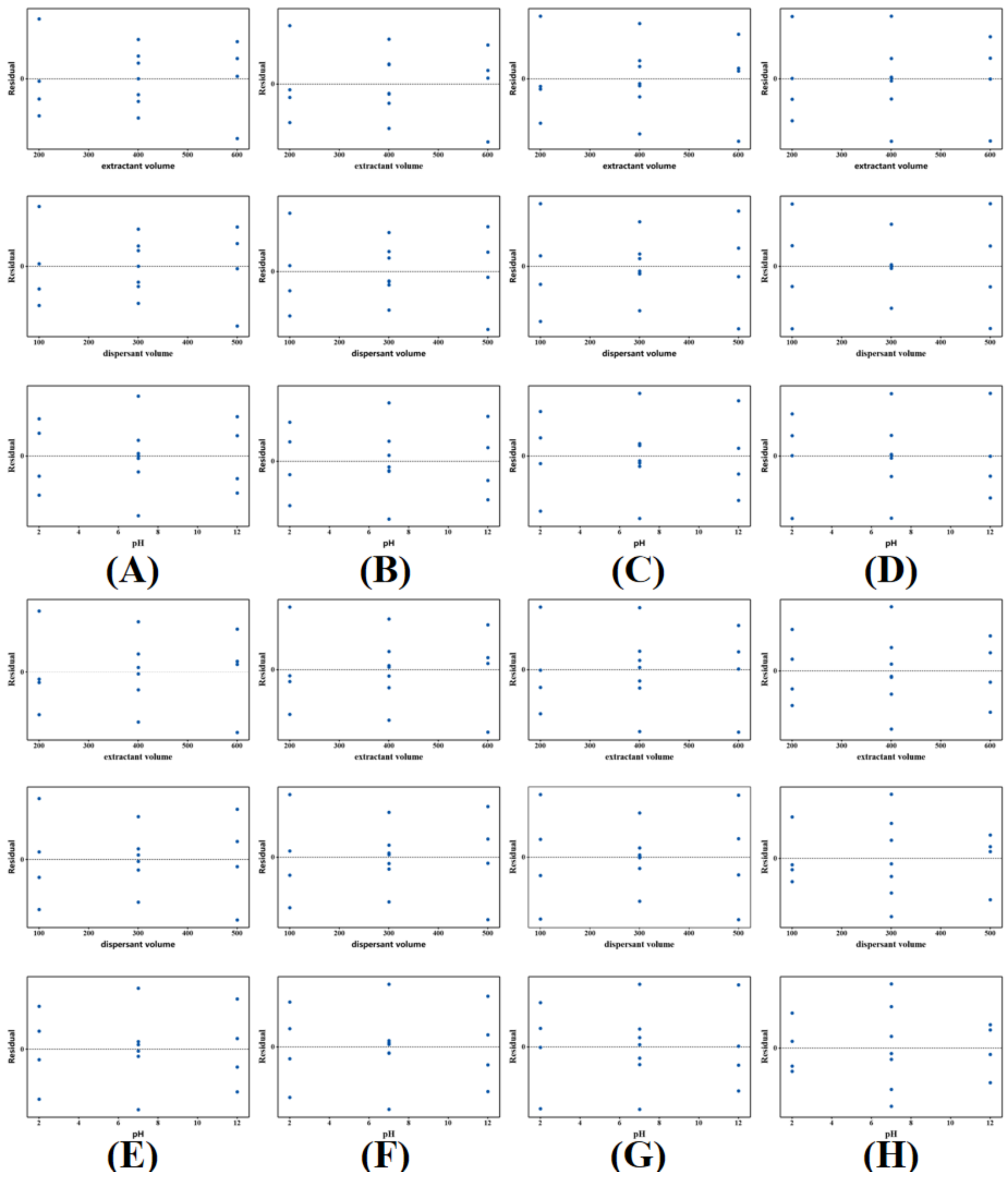

Fig. S2 Residual versus vs. factors plots: (A) Ace; (B) Flu; (C) Ant; (D) Phe; (E) Flt; (F) Pyr; (G) BaA; (H) BaP.
